# Prognostic Significance of Preoperative and Postoperative Complement C3 Depletion in Gastric Cancer: A Three-Year Survival Investigation

**DOI:** 10.1155/2017/2161840

**Published:** 2017-09-14

**Authors:** Jinning Ye, Yufeng Ren, Jianhui Chen, Wu Song, Chuangqi Chen, Shirong Cai, Min Tan, Yujie Yuan, Yulong He

**Affiliations:** ^1^Center of Gastrointestinal Surgery, The First Affiliated Hospital, Sun Yat-Sen University, Guangzhou, Guangdong Province, China; ^2^Center of Gastric Cancer, Sun Yat-Sen University, Guangzhou, Guangdong Province, China; ^3^Department of Radiation Oncology, The First Affiliated Hospital, Sun Yat-Sen University, Guangzhou, Guangdong Province, China

## Abstract

**Objectives:**

The role of complement system in predicting prognosis of gastric cancer (GC) remains obscured. This study aims to explore the incidence of complement C3 depletion and associated outcomes in GC patients.

**Methods:**

between August 2013 and December 2013, 106 patients with gastric adenocarcinoma were prospectively analyzed. Plasma levels of complement C3 and C4 were detected at baseline, one day before surgery, and postoperative day 3, respectively. Patients with low C3 levels (<0.75 mg/mL) were considered as having complement depletion (CD), while others with normal C3 levels were included as control. The 3-year overall survival (OS), disease-free survival (DFS), and other outcomes were compared between both groups, with the CD incidence explored meanwhile.

**Results:**

The CD incidence was 28.3% before surgery but increased to 37.7% after surgery. Preoperative CD was related to prolonged hospital stay (22.7 versus 19.2 day, *P* = 0.032) and increased postoperative complications (33.3% versus 14.5%, *P* = 0.030) and hospital costs (*P* = 0.013). Besides, postoperative C3 depletion was significantly associated with decreased 3-year OS (*P* = 0.022) and DFS (*P* = 0.003). Moreover, postoperative C3 depletion and advanced tumor stage were independent predictive factors of poor prognosis.

**Conclusions:**

Complement C3 depletion occurring in gastric cancer was associated with poor short-term and long-term outcomes.

## 1. Introduction 

 Gastric cancer (GC) is the third leading cause of cancer-associated death worldwide [1]. Worse still, GC is correlated to a higher health burden in several countries such as China and Japan, where it is the most common cancer in both sexes. To date, long-term outcomes of GC in China remain quite poor due to over 80% advanced tumor stage at diagnosis and rich diversity of treatment [2]. Early diagnosis by gastroscopy and appropriate surgical treatment are essential for improved prognosis in such population. In clinical practice, some patients with identical clinical stage of cancer experience various survival times, indicating differences in GC biology [3]. Therefore, researches for investigating heterogeneity in genetic susceptibility and demographic characteristics are definitely required to understand the mechanism of gastric carcinogenesis.

Epidemiological studies have identified several risk factors for development of GC, including* Helicobacter pylori (Hp)* infection, high oral intake of salt-preserved foods, long-term smoking habit, and pernicious anemia [4]. Those factors are commonly related to chronic inflammation around gastric epithelium and play roles in gastric carcinogenesis [5]. It has been recognized that chronic inflammation typically caused by* Hp* infection is involved in GC onset, promotion, and progression [6–9].

Complement system is a collection of over 30 serum proteins that are integral to inflammatory processes and innate immunity against exogenous infection [10]. Given the insidious links between complement system and inflammation, some complement components may play partial roles in inflammation-related carcinogenesis. A previous review has suggested that complement activation could enhance innate immunity against cancer through direct tumor cell lysis or complement-dependent cytotoxicity [11]. However, other studies have indicated that complement components C5a and C3a generated by complement activation could promote tumor growth and progression [12, 13]. Taken as a whole, complement system seems to have dual effects on the carcinogenesis process.

As known to all, complement C3 is the point of convergence for three complement activation pathways. The storage of C3 in human body is abundant; however, its concentration in serum can be easily affected by infection or inflammation. Our previous studies have found that complement C3 depletion due to extensive complement activation would depress immunity and predict a poor prognosis of severe intra-abdominal infection [14, 15]. Whether such complement depletion observed during exogenous infection also occurs in GC remains unknown. In addition, the predictive value of complement C3 depletion in GC patients is not completely clear. We herein perform a prospective study in Chinese population to illustrate our hypotheses that complement C3 depletion does develop in GC patients and links to poor clinical outcomes.

## 2. Patients and Methods

This was a prospective cohort study of patients with GC at a tertiary level teaching hospital in southern China. Patients with pathological diagnosis of gastric carcinoma were recruited between August 2013 and December 2013. The study protocol was approved by the Institutional Review Board of our hospital and registered in ClinicalTrials.gov (NCT02425930). Written informed consents were obtained from all participants prior to any treatment.

### 2.1. Patients

Within the study period, consecutive patients referred or admitted to our center were screened for eligibility criteria. Inclusion criteria included the following: (1) pathological diagnosis of gastric adenocarcinoma with clinical stages of I–III based on abdominal and pelvic computed tomography (CT), (2) indication for radical gastrectomy (total or subtotal) after a multidisciplinary team (MDT) meeting, with adjuvant chemotherapy considered as a routine plan for pathologic stage IIB and above, and (3) adult age between 18 and 75 years, with no limitation of gender.

Patients with the following medical conditions were excluded from this study: another primary malignancy, cardiopulmonary dysfunction, active uncontrolled infection or psychosis, and chronic inflammatory disease (tuberculosis, Crohn's disease, etc.) except for* Hp* infection; any abdominal emergency surgery due to tumor progression, history of major abdominal surgery within the last six months, or nonradical gastrectomy for palliative care; and long-term use of corticosteroids, insulin, oral antidiabetic drugs, or other drugs for obesity. Besides, those who recently received blood transfusion or continuous renal replacement therapy were also excluded from participation.

### 2.2. Clinical Management Schemes

As routinely practiced in China, surgical residents would interview patients and collect personal and clinical data, such as the period of chief complains, features of symptoms, percentage of weight loss and cachexia defined as the history of weight loss greater than 5% during the last three months, status of feeding, and past medical, drug, and family history. All enrolled patients would allocate a clinical tumor stage (cTNM) during the MDT meeting, which was usually held weekly on Monday morning. Of note, patients scheduled to receive neoadjuvant chemotherapy were not included in the current study. In general, patients had to wait 2–4 days after admission to receive an operation. After a curative resection, histology tumor stage (pTNM) was assessed by experienced gastrointestinal pathologists. All tumor stages were determined using the 7th edition of American Joint Committee on Cancer (AJCC) TNM staging system for gastric cancer [16].

Generally, surgical treatment, which was consisted of total or subtotal gastrectomy with R0 resection and lymphadenectomy, could be accomplished by open or laparoscopic techniques. The detailed surgical procedures were primarily determined by surgeon's preference. After a definitive surgery, adjuvant chemotherapy, with S-1 (60 mg/m^2^) and oxaliplatin (85 mg/m^2^) as a main regimen for at least eight cycles (SOX regimen), was initiated 3-4 weeks after surgery. Specifically, postoperative radiotherapy would be considered in patients with regional positive lymph nodes or suspicious resection margin on final pathology report.

As previously described, follow-up was typically every three months for the first year after surgery, every six months for the second year, and twice a year thereafter [17]. Similar follow-up visits took place with a medical oncologist or radiation oncologist. In our clinical practice, the abdominal and pelvic contrast-enhanced CT scan was routinely performed for tumor recurrence surveillance.

### 2.3. Complement Levels Monitor and Study Outcomes

Peripheral blood samples were routinely collected at baseline and one day before and three days after surgery, respectively. Plasma was obtained from the centrifugation (3000*g*, 20 min, 4°C) and immediately stored at −80°C until tested. As previously described, complement C3 and C4 levels were dynamically measured with ELISA method, with routine blood counts and biochemical analyses performed meanwhile. Patients were stratified into two groups according to preoperative levels of C3. In detail, patients, who had low levels of C3 both at admission and one day before surgery (cutoff value, 0.75 mg/mL), were assigned to complement depletion (CD) group [14]. The rest with normal C3 level at least one-shot test prior to surgery were assigned into control group. Of note, the incidence of complement C3 depletion was determined according to percentage of values below the lower reference limit (reference interval, 0.75–1.35 mg/mL).

The primary outcome of this study was the 3-year overall survival (OS) after a radical surgery. The secondary outcomes included the incidence of complement depletion, disease-free survival (DFS), incidence of postoperative complications, length of stay (LOS), and hospital expenditure. Meanwhile, several predictive factors (gender, age, body mass index, American Society of Anesthesia score, baseline C3 level, etc.) of poor prognosis in GC patients were detected using the Cox-regression analysis.

### 2.4. Statistical Analyses

 Provided that level of significance is 5%, to have 90% power to detect a hazard ratio (HR) of 0.5 between binary intervals of plasma C3 levels with assumed one standard deviation (SD), at least 30 deaths should be observed within the follow-up period [18]. Besides, given an estimate of 30% for 3-year OS and a 10% loss of follow-up, at least 80 GC patients should be included for this study.

Descriptive statistics were employed to present demographic characteristics and oncologic outcomes. Data were expressed as means ± SD if not otherwise indicated. Survival analysis was performed using the Kaplan-Meier method and the log-rank test. The OS was calculated from the date of definitive operation to death resulting from any cause. The DFS was defined as the time interval from surgery to confirmed recurrence, occurrence of a new primary cancer, or death from any cause. Student's *t-*test and Mann-Whitney *U* test were employed for continuous variables, whereas Fisher's* exact* test and Chi-square test were used for categorical variables. Cox proportional hazards models were utilized to calculate HRs with 95% confidence intervals (CIs) for the relationship between complement C3 depletion and 3-year OS or DFS. All data analyses were performed using IBM® SPSS® Statistics (Statistics. 23.0; Chicago, IL). In general, two-tailed tests were used, with *P* value < 0.05 considered statistically significant.

## 3. Results 

**Figure 1:**
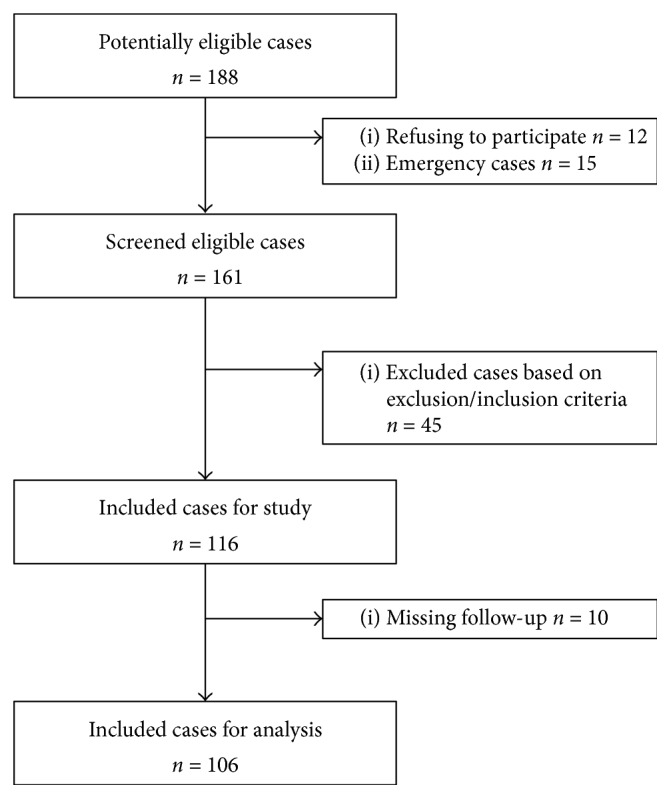
The enrollment and flowchart of current study population.

Between August 2013 and December 2013, 188 patients were enrolled for eligibility, and 106 patients were finally included based on inclusion and exclusion criteria (Figure 1). Of note, ten (8.6%) patients were lost during the follow-up period. The median follow-up period of this cohort was 23 (range, 1–40) months, with an observed death rate of 29.2% (31/106) after a radical gastrectomy. The demographic and baseline characteristics of included subjects were summarized in Table 1. Briefly, the median age of this cohort was 57 (range, 23–87) years, with 61.3% of male adults included. Among those, 30 (28.3%) patients suffering from preoperative C3 depletion were assigned into CD group, with the rest into control group. Although the case number in control group was twice of that in CD group, statistical comparisons in demographic and clinical data were not significant between both groups.

### 3.1. The Changes of Complement Components and Correlation Analyses

**Figure 2:**
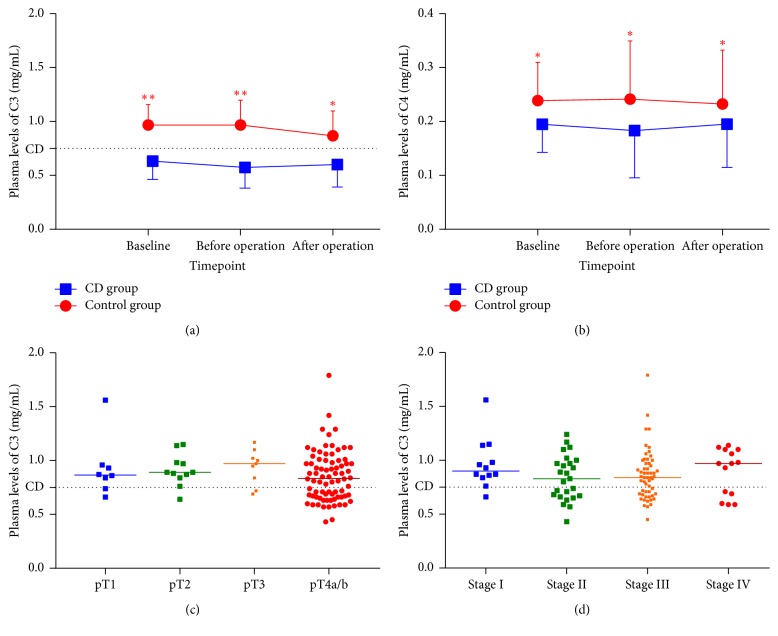
*The dynamic changes of complement components and baseline plasma levels of C3 in patients with gastric cancer*. Plasma levels of C3 (a) and C4 (b) within the perioperative period were quite different between CD group and control group (*P* < 0.05). However, the baseline levels of C3 had no significant differences among various invasive depths ((c), *P* = 0.561) or pathological stages ((d), *P* = 0.542). The extents of local tumor invasion are categorized into pTis, pT1, pT2, pT3, pT4a, and pT4b. The attached dotted line in each plot indicates reference boundary of complement C3 depletion. ^*∗∗*^*P* < 0.01, ^*∗*^*P* < 0.05 versus CD group.

As mentioned above, the function of complement system was mainly evaluated using changes of C3 levels at three time points: baseline at admission and one day before and three days after surgery. The average plasma levels of C3 and C4 at baseline were 0.87 (median, 0.87; range, 0.43–1.79) mg/mL and 0.23 (median, 0.22; range, 0.10–0.47) mg/mL, respectively. After surgery, both plasma levels declined in different degrees; however, such falling changes did not reach statistical significance compared to baseline values (*P* > 0.05). Besides, the number of patients suffering from CD increased from 30 (28.3%) at baseline to 40 (37.7%) after surgery, with 23 and 17 cases from control and CD group, respectively. Of 30 patients with preoperative CD, only 13 (43.3%) participants involving CD at first had improved plasma levels of complement C3 after surgery. There were significant differences in overall levels of C3 and C4 between CD group and control group (Figure 2). The current perioperative management for GC appeared to play minimal roles in enhancing complement function. Tumor invasive depth (*P* = 0.561) and pathological stage (*P* = 0.542) were not correlated to preoperative C3 depletion. In addition, body mass index (BMI), white blood cells (WBC) counts, hemoglobin, platelet (PLT) counts, plasma albumin, and total bilirubin (TB) were also not related to plasma C3 levels by a linear regression (Table 2). As a result, complement C3 depletion at admission or before surgery may be considered as an independent risk factor for predicting clinical outcomes of GC patients.

### 3.2. Primary and Secondary Outcomes

In the current study, 98 (92.5%) patients received open surgery for radical gastrectomy, with the rest undergoing laparoscopic surgery. Of note, intraoperative conversion due to massive hemorrhage was observed in one (12.5%) case from the CD group. Those secondary outcomes were shown in Table 3. The median LOS was 19 (range, 10–64) days, with nine (range, 3–52) days for length of postoperative stay (LOPS). Specifically, both LOS and LOPS were significantly prolonged in the CD group compared with the control group (*P* < 0.05). Moreover, the incidence of postoperative complications was markedly increased in the CD group compared with the control group (33.3% versus 14.5%, *P* = 0.030). In sum, 21 (19.8%) patients had various degrees of postoperative complications, with 12 for wound infection, 5 for postoperative pyrexia, 4 each for anastomotic leak and hemorrhage, and 3 for bowel obstruction. Among those, only two (9.5%) patients had to undergo unplanned reoperation for severe complications. However, no patient died in the hospital or within 30 days of operation. The total hospital expenditure was markedly increased in patients with preoperative CD compared to those without (*P* < 0.05).

**Figure 3:**
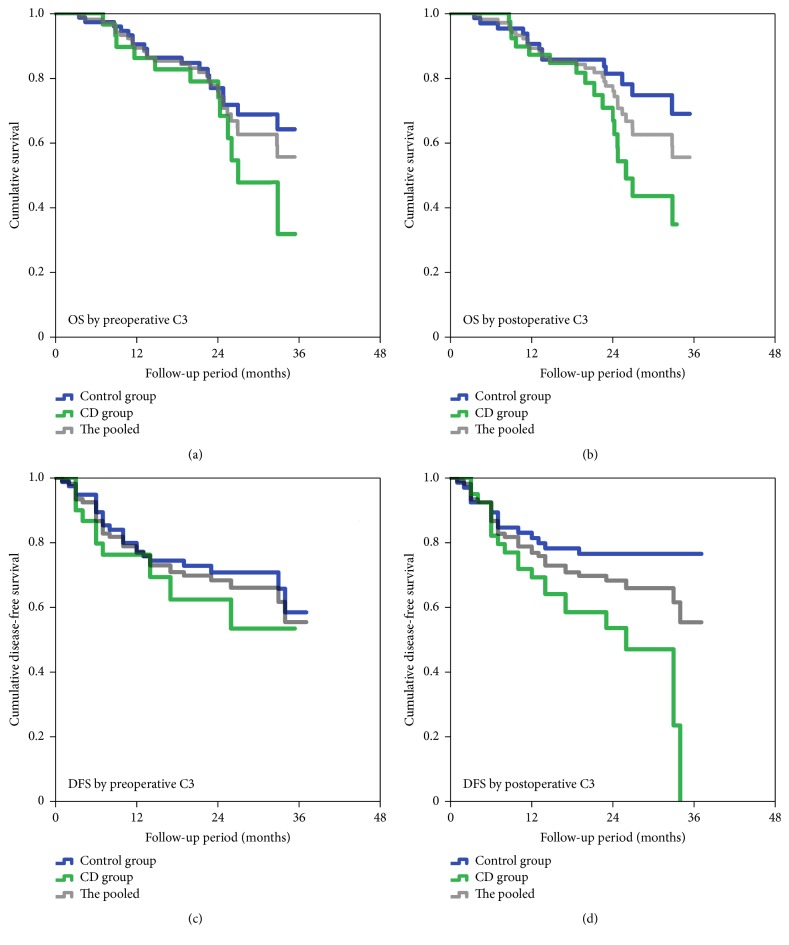
*Kaplan-Meier estimates of OS and DFS for GC patients by preoperative (a, c) and postoperative (b, d) plasma C3 levels (dichotomized by 0.75 mg/mL)*. The grey lines indicate the OS and DFS of included patients. CD group indicates patients with low plasma levels of C3, with control group for patients with normal plasma levels of C3.

Within the follow-up period, 31 (29.2%) patients died of tumor progression. Among those, 12 (38.7%) patients were in the CD group, with the others in the control group. The 3-year estimated OS rate was 51%, and the median estimated OS time for the current cohort was 26.0 (95% CI, 23.1–28.8) months. On the basis of Kaplan-Meier survival analysis, the OS time had no significant difference between both groups (*P* = 0.162, Figure 3). However, patients who developed postoperative CD would have much poorer OS than those without postoperative CD (*P* = 0.022, Figure 3). On the other hand, 35 (33%) patients developed tumor recurrence within the median follow-up period of 20.9 (range, 1–37) months. Similarly, Kaplan-Meier estimates of DFS by baseline C3 levels had no significant difference (*P* = 0.258, Figure 3), while the DFS by postoperative C3 levels appeared statistically significant (*P* = 0.003). A Cox regression using univariate analysis indicated that postoperative C3 levels, pathological T stage, N stage, and total tumor stage (pTNM), and tumor marker were associated with long-term survival (Table 4). A further analysis with stepwise multivariate regression revealed that postoperative C3 level and pTNM were independent predictive factors of poor survival in such cohort. Specifically, postoperative decreased C3 levels (<0.75 mg/mL) and advanced tumor stage (stage III/IV) were correlated with a poor prognosis for GC patients.

## 4. Discussion

In the current study, 106 patients with resectable gastric cancer were prospectively followed. To our knowledge, the incidence of complement depletion for such patients was 28.3% in the beginning but increased to 37.7% after a definitive operation. Our results indicated that preoperative complement depletion was an independent risk factor during preoperative evaluation. It was associated with prolonged stay in hospital and increased postoperative complications and hospital expenses. Importantly, the 3-year OS and DFS were both decreased in patients with CD compared to those without CD. Besides, we found that postoperative CD, rather than preoperative CD, had prognostic significance for those long-term outcomes. By using Cox logistic regression analyses, postoperative CD and advanced tumor stage were independent predictive factors of a poor prognosis.

As known to all, complement system is an old member of innate immunity, which plays key roles in defending against exogenous pathogens and regulating intrinsic inflammation [15]. Its biological functions, producing numerous active effectors for immunity regulation and opsonization, are realized majorly through three activation pathways: classical, lectin, and alternative ways [19]. Nowadays, more and more evidence have demonstrated that such pathways are minutely regulated based on various plasma levels of complement components, particularly complement C3 [20]. Our previous studies have found that complement C3 exhaustion due to severe sepsis would deteriorate immunity and coagulopathy meanwhile [14, 15].

While there is great interest in complement system in various inflammation-related diseases, there have been limited studies that focus on the exhaustion of complement by-products and subsequent outcomes in cancer diseases. A recent literature review indicates that complement system definitely plays roles in carcinogenesis of certain cancers, such as bladder, cervical, and hepatocellular carcinomas [21]. To our knowledge, the current study first reported the incidence of complement depletion and its predictive value for short-term and long-term outcomes in GC.

In clinical practice, complement dysfunction is commonly underestimated or even ignored during the preoperative evaluation, as compared with tumor-related obstruction and hemorrhage, malnutrition, and coagulopathy. Hence, its role in predicting postoperative outcomes, particularly long-term results, would be completely unclear. Our study has provided a referenced CD incidence (28.3%–37.7%) in GC patients but, more importantly, offered a hint to alert complement dysfunction in the perioperative management of gastric cancer.

Based on our preliminary studies, it is believed that persistent CD due to severe intra-abdominal infection was related to a poor prognosis. Similarly, it is confirmed that postoperative CD in GC patients was associated with poor OS and DFS. However, the concrete mechanism of CD-associated cancer surveillance remains obscured. To our knowledge, the vast majority of gastric cancers grow for years and induce repeatedly chronic inflammation. Therefore, uninterrupted complement activation would be inevitable, and circulatory complement insufficiency would be predictable. In spite of intrinsic consumption, complement proteins, such as C3d, C5b-9, and S protein, would be deposited on GC cells to avoid complement-mediated cytotoxicity [22]. These possible pathways for consuming complement components might contribute to the observed CD in GC patients. On the other hand, a depressed complement activity could indirectly promote the growth of gastric cancer cells and allow distant metastasis due to resistance to immune attack [23–25]. However, it is still unknown why only a few patients would develop CD in clinical practice of GC treatment. A recent study indicates that a certain genotype of type I complement receptor (CR) is highly expressed in Chinese population and strongly associated with decreased complement activation and reduced GC risk [10].

Several limitations of this study should be addressed. First, the small sample size due to relatively short recruiting period might depress statistical power and conceal some significances of current findings. Given limited number of patients with early-stage disease, a subgroup analysis by tumor stage was failed to perform in this study. Second, other complement proteins, such as C3a, C5a, type I CR, and factor B, were not available for daily clinical tests. Exploring plasma changes of those effectors in GC will enhance our understanding of roles of complement system in cancer surveillance and inflammation. Indeed, the complement C3 depletion was not fully equal with the complement depletion, since other activation pathways did work independently without complement C3. At last, future studies with larger samples and focus on the molecular mechanism of CD-associated cancer surveillance are needed to answer the following question: “is complement good or bad for cancer patients?”

In summary, our results demonstrate that complement C3 depletion could indicate poor clinical outcomes for gastric cancer patients in China. Additionally, such depletion emerging after a definitive operation seems to be an independent predictive factor of poor long-term survival. The complement C3 depletion, whether before or after surgery, should be paid close attention in clinical surveillance of this subset population.

## Figures and Tables

**Table 1:** The demographic and baseline characteristics of patients with gastric cancer.

	The pooled	CD group	Control group	*P* value
	(*n* = 106)	(*n* = 30)	(*n* = 76)	
Age, yrs	56.4 ± 12.2	54.5 ± 15.1	57.2 ± 10.9	0.370
≤65	82 (77.4)	20 (66.7)	62 (81.6)	0.084
>65	24 (22.6)	10 (33.3)	14 (18.4)	
Gender, male : female	65 : 41	16 : 14	49 : 27	0.376
BMI, kg/m^2^	21.6 ± 3.3	20.9 ± 2.8	21.8 ± 3.4	0.231
Comorbidity, *n* (%)				
HTN	9 (8.5)	3 (10.0)	6 (7.9)	0.710
DM	4 (3.8)	2 (6.7)	2 (2.6)	0.317
Smoke, *n* (%)	26 (24.5)	9 (30.0)	17 (22.4)	0.456
Alcohol abuse, *n* (%)	13 (12.3)	11 (14.5)	2 (6.7)	0.342
ASA, *n* (%)				0.594
I + II	85 (80.2)	23 (76.7)	62 (81.6)	
≥III	21 (19.8)	7 (23.3)	14 (18.4)	
Albumin level, g/dL	38.1 ± 7.5	37.9 ± 4.6	38.2 ± 8.4	0.880
Operative time, min	278.7 ± 78.4	281.8 ± 71.8	277.4 ± 81.4	0.796
Type of anastomosis, *n* (%)				0.661
Billroth I	2 (1.9)	0	2 (2.6)	
Billroth II	11 (10.4)	3 (10.0)	8 (10.5)	
Roux-en-Y	51 (48.1)	15 (50.0)	36 (47.4)	
Gastrectomy technique, *n* (%)				0.803
Proximal gastrectomy	13 (12.3)	3 (10.0)	10 (13.2)	
Distal gastrectomy	45 (42.4)	12 (40.0)	33 (43.4)	
Total gastrectomy	48 (45.3)	15 (50.0)	33 (43.4)	
Lymphadenectomy, *n* (%)				0.566
*D*_1_	8 (7.5)	1 (3.3)	7 (9.2)	
*D*_2_	76 (71.7)	23 (76.7)	53 (69.7)	
*D*_2_ plus^*∗*^	22 (20.8)	6 (20.0)	16 (21.1)	
Depth of invasion, *n* (%)				0.251
pT1	8 (7.6)	1 (3.3)	7 (9.2)	
pT2	11 (10.3)	1 (3.3)	10 (13.1)	
pT3	9 (8.5)	2 (6.7)	7 (9.2)	
pT4	78 (73.6)	26 (86.7)	52 (68.4)	
Lymph nodes retrievals, *n*	39.2 ± 18.8	41.9 ± 18.4	38.1 ± 19.0	
Lymph nodes metastases, *n* (%)				0.650
pN0	31 (29.2)	11 (36.7)	21 (27.6)	
pN1	14 (13.2)	3 (10.0)	11 (14.5)	
pN2	19 (17.9)	4 (13.3)	16 (21.1)	
pN3	40 (37.7)	12 (40.0)	28 (36.8)	
Histopathological type, *n* (%)				0.978
Well diff.	7 (6.6)	2 (6.7)	5 (6.5)	
Moderate diff.	19 (17.9)	5 (16.7)	14 (18.4)	
Poor diff.	80 (75.5)	23 (76.6)	57 (75.0)	
pTNM Stage, *n* (%)				0.223
I	12 (11.3)	1 (3.3)	11 (14.5)	
II	25 (23.6)	10 (33.3)	15 (19.7)	
III	55 (51.9)	16 (53.4)	39 (51.3)	
IV	14 (13.2)	3 (10.0)	11 (14.5)	

Patients were divided into CD group and control group based on baseline levels of C3 (cutoff value, 0.75 mg/mL). Data present with mean ± SD or number (percentage of column). CD, complement depletion; BMI, body mass index; HTN, hypertension; DM, diabetes mellitus; ASA, American Society of Anesthesia; diff., differentiation.  ^*∗*^*D*_2_ plus means extended lymphadenectomy beyond *D*_2_ stations during gastrectomy.

**Table 2:** Univariate linear regression between C3 and general factors.

Variable	*R*	*B*	SE	*P* value
BMI, kg/m^2^	0.177	0.012	0.007	0.070
WBC, ×10^9^/L	0.017	0.002	0.011	0.861
HB, ×10^9^/L	0.066	−0.001	0.001	0.498
PLT, ×10^9^/L	0.067	0.001	0.001	0.494
Albumin, g/dl	0.004	0.001	0.003	0.967
TB, mg/mL	0.109	0.005	0.005	0.267

BMI, body mass index; WBC, white blood cells; HB, hemoglobin; PLT, platelets; TB, total bilirubin; *R*, correlation coefficient; *B*, slope of the regression (also known as regression coefficient); SE, standard errors. *P* < 0.05 considered statistically correlated between C3 and factor.

**Table 3:** The short-term outcomes after a definitive operation for gastric cancer.

Parameter	The pooled (*n* = 106)	CD group (*n* = 30)	Control group (*n* = 76)	*P* value
LOS, day	20.2 ± 7.57	22.7 ± 7.5	19.2 ± 7.4	0.032
LOPS, day	10.8 ± 6.4	12.8 ± 5.9	10.1 ± 6.5	0.025
Hospital expenses, rmb	73,484 ± 31,337	85,385 ± 30,686	68,786 ± 30,523	0.013
Postoperative complications, *n* (%)	21 (19.8)	10 (33.3)	11 (14.5)	0.030
Pyrexia	5 (4.7)	1 (3.3)	4 (5.3)	
SSI	12 (11.3)	5 (16.7)	7 (9.2)	
Bowel obstruction/ileus	3 (2.8)	1 (3.3)	2 (2.6)	
Anastomotic leak	4 (3.8)	2 (6.7)	2 (2.6)	
IAH	4 (3.8)	3 (10.0)	1 (1.3)	

LOS, length of stay; LOPS, length of postoperative stay; SSI, surgical site infection; IAH, intra-abdominal hemorrhage.

**Table 4:** Univariate and multivariate analyses for predicative factors of poor prognosis in gastric cancer patients.

Variable	Univariate analysis				Multivariate analysis		
	*B*	HR	95% CI of HR	*P* value	HR	95% CI of HR	*P* value
Age	−0.212	0.809	0.399–1.642	0.558			
Gender	0.230	1.258	0.579–2.735	0.562			
BMI	−0.413	0.662	0.285–1.539	0.338			
Tumor Heredity	0.435	1.546	0.449–5.318	0.490			
HTN	−1.102	0.332	0.045–2.469	0.282			
DM	−0.155	0.857	0.110–6.641	0.882			
Smoke	0.542	1.720	0.768–3.849	0.187			
Alcohol	−0.114	0.893	0.291–2.736	0.842			
Baseline C3	0.536	0.709	0.828–3.529	0.148			
Postoperative C3	0.833	2.300	1.131–4.677	0.022	2.640	1.263–5.518	0.010
Tumor location	−0.014	0.986	0.883–1.101	0.804			
pT stage	1.370	3.934	1.256–12.326	0.019	2.386	0.720–7.911	0.155
pN stage	−2.257	0.105	0.029–0.377	0.001	1.532	0.964–2.433	0.071
pTNM stage	1.622	5.065	2.698–9.509	0.001	1.749	2.024–11.141	0.001
Histology	0.372	1.450	0.760–2.769	0.260			
Tumor marker^*∗*^	0.620	1.860	1.229–2.814	0.003	0.867	0.515–1.462	0.593

Cox regression method was utilized for those analyses, with the following cutoff values for risk factors: age, 65 yrs; BMI, 18.5 kg/m^2^; tumor heredity, GC family history; HTN, hypertension; DM, diabetes mellitus; C3, 0.75 mg/mL at admission or postoperative day 3; tumor location, up/middle/low 1/3 stomach; TNM stage, I/II versus III/IV; histology, high/moderate/low differentiation; tumor marker, any increased values beyond upper limit of reference range at admission. Multivariate analysis using postoperative C3, pT stage, pN stage, pTNM stage, and tumor marker was performed with Enter mode. ^**∗**^tumor makers in digestive malignant diseases, including AFP (0–20 *μ*g/L), CEA (0–5 *μ*g/L), CA125 (0–35 U/mL), CA19-9 (0–35 U/mL), and SCC (0–1.5 *μ*g/L), were measured at admission or follow-up visits.

## References

[1] Siegel R. L., Miller K. D., Jemal A. (2015). Cancer statistics, 2015. *CA: Cancer Journal for Clinicians*.

[2] Zong L., Abe M., Seto Y., Ji J. (2016). The challenge of screening for early gastric cancer in China. *The Lancet*.

[3] Sawada T., Yashiro M., Sentani K. (2015). New molecular staging with G-factor supplements TNM classification in gastric cancer: a multicenter collaborative research by the Japan Society for Gastroenterological Carcinogenesis G-Project committee. *Gastric Cancer*.

[4] Guggenheim D. E., Shah M. A. (2013). Gastric cancer epidemiology and risk factors. *Journal of Surgical Oncology*.

[5] Uemura N., Okamoto S., Yamamoto S. (2001). Helicobacter pylori infection and the development of gastric cancer. *The New England Journal of Medicine*.

[6] Li G., Wang Z., Ye J. (2014). Uncontrolled inflammation induced by AEG-1 promotes gastric cancer and poor prognosis. *Cancer Research*.

[7] Li G., Wang Z., Wang Z. (2013). Gastric cancer patients with Helicobacter pylori infection have a poor prognosis. *Journal of Surgical Oncology*.

[8] Wang F., Meng W., Wang B., Qiao L. (2014). Helicobacter pylori-induced gastric inflammation and gastric cancer. *Cancer Letters*.

[9] Epplein M., Xiang Y.-B., Cai Q. (2013). Circulating cytokines and gastric cancer risk. *Cancer Causes and Control*.

[10] Zhao L., Zhang Z., Lin J. (2015). Complement receptor 1 genetic variants contribute to the susceptibility to gastric cancer in Chinese population. *Journal of Cancer*.

[11] Gelderman K. A., Tomlinson S., Ross G. D., Gorter A. (2004). Complement function in mAb-mediated cancer immunotherapy. *Trends in Immunology*.

[12] Markiewski M. M., DeAngelis R. A., Benencia F. (2008). Modulation of the antitumor immune response by complement. *Nature Immunology*.

[13] Ostrand-Rosenberg S. (2008). Cancer and complement. *Nature Biotechnology*.

[14] Ren J., Zhao Y., Yuan Y. (2012). Complement depletion deteriorates clinical outcomes of severe abdominal sepsis: a conspirator of infection and coagulopathy in crime?. *PLoS ONE*.

[15] Yuan Y., Yan D., Han G., Gu G., Ren J. (2013). Complement C3 depletion links to the expansion of regulatory T cells and compromises T-cell immunity in human abdominal sepsis: a prospective pilot study. *Journal of Critical Care*.

[16] Washington K. (2010). 7th edition of the AJCC cancer staging manual: stomach. *Annals of Surgical Oncology*.

[17] Song W., Yuan Y., Wang L. (2014). The prognostic value of lymph nodes dissection number on survival of patients with lymph node-negative gastric cancer. *Gastroenterology Research and Practice*.

[18] Schoenfeld D. A. (1983). Sample-size formula for the proportional- hazards regression model.. *Biometrics*.

[19] Ricklin D., Lambris J. D. (2013). Complement in immune and inflammatory disorders: pathophysiological mechanisms. *Journal of Immunology*.

[20] Delanghe J. R., Speeckaert R., Speeckaert M. M. (2014). Complement C3 and its polymorphism: Biological and clinical consequences. *Pathology*.

[21] Markiewski M. M., Lambris J. D. (2009). Is complement good or bad for cancer patients? A new perspective on an old dilemma. *Trends in Immunology*.

[22] Inoue T., Yamakawa M., Takahashi T. (2002). Expression of complement regulating factors in gastric cancer cells. *Journal of Clinical Pathology - Molecular Pathology*.

[23] Fishelson Z., Donin N., Zell S., Schultz S., Kirschfink M. (2003). Obstacles to cancer immunotherapy: Expression of membrane complement regulatory proteins (mCRPs) in tumors. *Molecular Immunology*.

[24] Donin N., Jurianz K., Ziporen L., Schultz S., Kirschfink M., Fishelson Z. (2003). Complement resistance of human carcinoma cells depends on membrane regulatory proteins, protein kinases and sialic acid. *Clinical and Experimental Immunology*.

[25] Wilczek E., Rzepko R., Nowis D. (2008). The possible role of factor H in colon cancer resistance to complement attack. *International Journal of Cancer*.

